# P-2087. Sociodemographic Factors Associated with Adverse Outcomes in Patients with Pneumonia

**DOI:** 10.1093/ofid/ofaf695.2251

**Published:** 2026-01-11

**Authors:** Katherine Moore, Ryan Moazamian, Ashley Amundson, Henry S Fraimow, Carlo Foppiano Palacios

**Affiliations:** Cooper Medical School of Rowan University, Lancaster, PA; Cooper University Hospital, Camden, New Jersey; Rutgers New Jersey Medical School, Newark, New Jersey; Cooper University Hospital and Cooper Medical School of Rowan University, Camden, NJ; Cooper University Health Care, Camden, New Jersey

## Abstract

**Background:**

Pneumonia is one of the leading causes of morbidity and mortality worldwide. During the COVID-19 pandemic, we learned the impact of social determinants of health (SDH) on patient outcomes; however, their impact on non-COVID-19 pneumonia is unclear. We aim to evaluate the association between SDH and clinical outcomes in patients with non-COVID-19 pneumonia.

**Methods:**

We conducted a single-center, retrospective chart review from August 2021 to December 2021 at Cooper University Hospital, an academic-tertiary medical center. We included all patients admitted to the hospital with ICD-code J18.9 and excluded patients with COVID-19. We collected demographic data including census-block level measures of vulnerability, culture results, treatments, discharge status, and outcomes at 30 and 90 days. Additionally, patients’ address were used to calculate census-tract level data associated with American Community Survey, Area Deprivation Index (ADI: ranked on a scale 1 to 10, with 10 being most disadvantaged), and Social Vulnerability Index (SVI: ranked on a scale of 0 to 1, with 1 being most vulnerable). Descriptive statistics were performed and Kruskal Wallis and Fisher’s exact testing for bivariate analyses.

**Results:**

143 pneumonia episodes among 137 patients were included: mean age was 57 years, 50% were male, 45% were White, and 17% were Latino. Patients lived in vulnerable areas: mean ADI index was 8.9, mean SVI index was 0.7. SDH were prevalent in patients admitted for pneumonia: 31% had substance abuse history, 23% had mental health history, 23% of patients did not speak English, and 7% had housing insecurity. Sixty-eight (49.6%) patients had at least one of these variables. Mean hospital length of stay was 7.7 days, and 6% of episodes resulted in mortality. We found no association between social determinants of health and mortality or readmission outcomes at hospital discharge, and 30- or 60-days post-discharge.

**Conclusion:**

While we found no association between sociodemographic factors and mortality in our limited dataset, our study population faced significant SDH. Future analysis should evaluate the relationship between SDH and the evaluation, diagnosis, decision to hospitalize, and treatment in patients presenting with suspected pneumonia using a larger subset of data.

**Disclosures:**

All Authors: No reported disclosures

